# Complete genome sequence of *Thermaerobacter marianensis* type strain (7p75a^T^)

**DOI:** 10.4056/sigs.1373474

**Published:** 2010-12-15

**Authors:** Cliff Han, Wei Gu, Xiaojing Zhang, Alla Lapidus, Matt Nolan, Alex Copeland, Susan Lucas, Tijana Glavina Del Rio, Hope Tice, Jan-Fang Cheng, Roxane Tapia, Lynne Goodwin, Sam Pitluck, Ioanna Pagani, Natalia Ivanova, Konstantinos Mavromatis, Natalia Mikhailova, Amrita Pati, Amy Chen, Krishna Palaniappan, Miriam Land, Loren Hauser, Yun-Juan Chang, Cynthia D. Jeffries, Susanne Schneider, Manfred Rohde, Markus Göker, Rüdiger Pukall, Tanja Woyke, James Bristow, Jonathan A. Eisen, Victor Markowitz, Philip Hugenholtz, Nikos C. Kyrpides, Hans-Peter Klenk, John C. Detter

**Affiliations:** 1 DOE Joint Genome Institute, Walnut Creek, California, USA; 2 Los Alamos National Laboratory, Bioscience Division, Los Alamos, New Mexico, USA; 3 Biological Data Management and Technology Center, Lawrence Berkeley National Laboratory, Berkeley, California, USA; 4 Oak Ridge National Laboratory, Oak Ridge, Tennessee, USA; 5 DSMZ - German Collection of Microorganisms and Cell Cultures GmbH, Braunschweig, Germany; 6 HZI – Helmholtz Centre for Infection Research, Braunschweig, Germany; 7 University of California Davis Genome Center, Davis, California, USA

**Keywords:** strictly aerobic, none-motile, Gram-variable, thermophilic, chemoheterotrophic, deep-sea, family Incertae Sedis XVII, *Clostridiales*, GEBA

## Abstract

*Thermaerobacter marianensis* Takai *et al.* 1999 is the type species of the genus *Thermaerobacter*, which belongs to the *Clostridiales* family Incertae Sedis XVII. The species is of special interest because *T. marianensis* is an aerobic, thermophilic marine bacterium, originally isolated from the deepest part in the western Pacific Ocean (Mariana Trench) at the depth of 10.897m. Interestingly, the taxonomic status of the genus has not been clarified until now. The genus *Thermaerobacter* may represent a very deep group within the *Firmicutes* or potentially a novel phylum. The 2,844,696 bp long genome with its 2,375 protein-coding and 60 RNA genes consists of one circular chromosome and is a part of the *** G****enomic* *** E****ncyclopedia of* *** B****acteria and* *** A****rchaea * project.

## Introduction

Strain 7p75a^T^ (= DSM 12885 = ATCC 700841 = JCM 10246) is the type strain of *T. marianensis* which is the type species of the genus *Thermaerobacter* [1,2]. Currently, there are five species placed in the genus *Thermaerobacter* [1,3]. The generic name derives from the Greek words ‘*thermos*’ meaning ‘hot’, ‘*aeros*’(air) and the Neo-Latin word ‘*bacter*’ meaning ‘a rod’, which altogether means able to grow at high temperatures in the presence of air [2]. The species epithet is derived from the Neo-Latin word ‘*marianensis*’ pertaining to the Mariana Trench, the location from which the strain was isolated from [2]. *T. marianensis* strain 7p75a^T^ was isolated from a mud sample of the Challenger Deep in the Mariana Trench at the depth of 10,897 m [2]. No further isolates have been obtained for *T. marianensis*. Other members of the genus *Thermaerobacter* were isolated from mud of the bottom of the Challenger Deep [2], shallow marine hydrothermal vent, Japan [4], water sediment slurries of the run-off channel of New Lorne Bore, Australia [5], a coastal hydrothermal beach, Japan [6] and from food sludge compost, Japan [7]. Here we present a summary classification and a set of features for *T. marianensis* 7p75a^T^, together with the description of the complete genomic sequencing and annotation.

## Classification and features

The 16S rRNA gene sequences of *T. marianensis* 7p75a^T^ share 98.3 to 98.6% sequence identity with the other type strains of the genus *Thermaerobacter* [2,8] and *T. nagasakiensis* being the closest relative. Outside the genus members of the recently proposed genus “*Calditerricola”* (88.6%) [9] and the genus *Moorella* (88.1%) [10] share the highest degree of sequence similarity. The genomic survey sequence database (gss) contains as best hits several 16S rRNA gene sequence from The Sorcerer II Global Ocean Sampling Expedition: Northwest Atlantic through Eastern Tropical Pacific [11] at a similarity level of only 84%. No phylotypes from environmental samples database (env_nt) could be linked to the species *T. marianensis* or even the genus *Thermaerobacter*, indicating a rather rare occurrence of members of this genus in the habitats screened thus far (as of November 2010).

A representative genomic 16S rRNA sequence of *T. marianensis* 7p75a^T^ was compared using NCBI BLAST under default values (e.g., considering only the best 250 hits) with the most recent release of the Greengenes database [12] and the relative frequencies of taxa and keywords, weighted by BLAST scores, were determined. The five most frequent genera were *Thermaerobacter* (39.7%), *Moorella* (35.3%), *Geobacillus* (6.4%), *Thermoactinomyces* (3.6%) and *Bacillus* (3.4%). Regarding hits to sequences from other members of the genus, the average identity within HSPs (high-scoring segment pairs) was 98.1%, whereas the average coverage by HSPs was 96.3%. The species yielding the highest score was *Thermaerobacter subterraneus*. The five most frequent keywords within the labels of environmental samples which yielded hits were 'compost(ing)' (6.1%), 'municipal' (2.9%), 'scale' (2.7%) and 'process/stages' (2.6%). Environmental samples which yielded hits of a higher score than the highest scoring species were not found.

**Figure 1:**
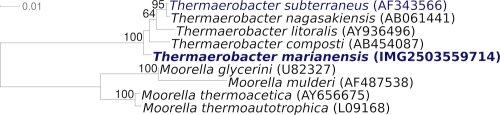
Phylogenetic tree highlighting the position of *T. marianensis* 7p75a^T^ relative to the other type strains within the family. The tree was inferred from 1,489 aligned characters [13,14] of the 16S rRNA gene sequence under the maximum likelihood criterion [15] and rooted in accordance with the current taxonomy. The branches are scaled in terms of the expected number of substitutions per site. Numbers above branches are support values from 1,000 bootstrap replicates [16] if larger than 60%. Lineages with type strain genome sequencing projects registered in GOLD [17] are shown in blue, published genomes in bold.

Figure 1 shows the phylogenetic neighborhood of *T. marianensis* 7p75a^T^ in a 16S rRNA based tree. The sequences of the two 16S rRNA gene copies differ from each other by two nucleotides, and differ by up to two nucleotides from the previously published 16S rRNA sequence (AB011495), which contains one ambiguous base call.

**Figure 2:**
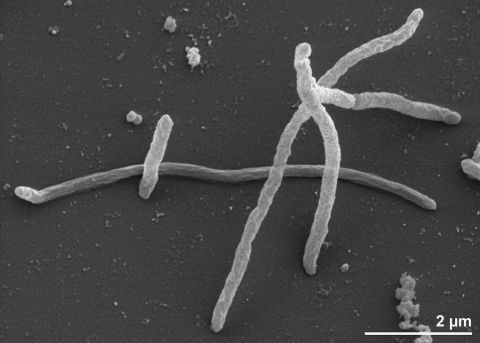
Scanning electron micrograph of *T. marianensis* 7p75a^T^

The cells of *T. marianensis* are generally rod-shaped (0.3-0.6 × 2-7 µm), straight to slightly curved with rounded ends (Figure 2). The cells can be arranged in pairs [2]. *T. marianensis* is a Gram-positive, spore-forming bacterium (Table 1). At stationary phase cells, may stain Gram-negative. Motility and flagella have not been observed [2], but genes for biosynthesis and assembly of flagella have been identified in the here reported genome sequence. The organism is a strictly aerobic chemoheterotroph. *T. marianensis* is a typical marine bacterium and requires sea salts (0.5-5%, optimum 2%) in media for good growth [2]. The temperature range for growth is between 50°C and 80°C, with an optimum at 75°C [2]. The pH range for growth is 5.4-9.5, with an optimum at pH 7.0-7.5 [2]. *T. marianensis* is able to grow on yeast extract, peptone and casein. It utilizes carbohydrates like starch, xylan, chitin, maltose, maltotriose, cellobiose, lactose, trehalose, sucrose, glucose, galactose, xylose, mannitol, inositol. The strain is also able to grow on amino acids like casamino acids, valine, isoleucine, cysteine, proline, serine, threonine, asparagine, glutamine, aspartate, glutamate, lysine, arginine and histidine. *T. marianensis* is able to grow well on various carboxylic acids like propionate, 2-aminobutyric acid, malate, pyruvate, tartarate, succinate, lactate, acetate and glycerol [2]

**Table 1:** Classification and general features of *T. marianens* 7p75a^T^ according to the MIGS recommendations [18].

MIGS ID	Property	Term	Evidence code
	Current classification	Domain *Bacteria*	TAS [19]
		Phylum *Firmicutes*	TAS [20-22]
		Class *Clostridia*	TAS [20,23,24]
		Order *Clostridiales*	TAS [20,25,26]
		Family Incertae sedis XVII	TAS [20]
		Genus *Thermaerobacter*	TAS [2,5,27]
		Species *Thermaerobacter marianensis*	TAS [2,27]
		Type strain 7p75a	TAS [2]
	Gram stain	variable, slightly Gram-positive	TAS [2]
	Cell shape	straight to slightly rods with rounded ends, singly or in pairs	TAS [2]
	Motility	non-motile	TAS [2]
	Sporulation	terminal, round spores (rarely detectable)	IDA
	Temperature range	50°C-80°C	TAS [2]
	Optimum temperature	75	TAS [2]
	Salinity	requirement for sea salts (0.5-5%)	TAS [2]
MIGS-22	Oxygen requirement	strictly aerobic	TAS [2]
	Carbon source	carbohydrates	TAS [2]
	Energy source	chemoheterotrophic	TAS [2]
MIGS-6	Habitat	mud	TAS [2]
MIGS-15	Biotic relationship	free-living	NAS
MIGS-14	Pathogenicity	none	NAS
	Biosafety level	1	TAS [28]
	Isolation	marine mud sample	TAS [2]
MIGS-4	Geographic location	Challenger Deep; Mariana Trench	TAS [2]
MIGS-5	Sample collection time	1996	TAS [2]
MIGS-4.1	Latitude	11.35	TAS [2]
MIGS-4.2	Longitude	142.41	TAS [2]
MIGS-4.3	Depth	10,897 m	TAS [2]
MIGS-4.4	Altitude	-10,897 m	NAS

Evidence codes - IDA: Inferred from Direct Assay (first time in publication); TAS: Traceable Author Statement (i.e., a direct report exists in the literature); NAS: Non-traceable Author Statement (i.e., not directly observed for the living, isolated sample, but based on a generally accepted property for the species, or anecdotal evidence). These evidence codes are from of the Gene Ontology project [29]. If the evidence code is IDA, then the property was directly observed by one of the authors or an expert mentioned in the acknowledgements.

### Chemotaxonomy

The cellular polyamines of the strain 7p75a^T^ were identified as N4-bis(aminopropyl)spermidine, agmatine, spermidine, and spermine [30,31]. The major cellular fatty acids were composed of 15-methyl-hexadecanic acid (52.3%), myristoleic acid (27.6%) and 14-methyl hexadecanoic acid (9.3%) [2]. No data are available for polar lipids and peptidoglycan type of the cell wall.

## Genome sequencing and annotation

### Genome project history

This organism was selected for sequencing on the basis of its phylogenetic position [32], and is part of the *** G****enomic* *** E****ncyclopedia of* *** B****acteria and* *** A****rchaea * project [33]. The genome project is deposited in the Genome OnLine Database [17,34] and the complete genome sequence is deposited in GenBank. Sequencing, finishing and annotation were performed by the DOE Joint Genome Institute (JGI). A summary of the project information is shown in Table 2.

**Table 2:** Genome sequencing project information

**MIGS ID**	**Property**	**Term**
MIGS-31	Finishing quality	Finished
MIGS-28	Libraries used	Three genomic libraries: one 454 pyrosequence standard library, one 454 PE library (9 kb insert size), one Illumina library
MIGS-29	Sequencing platforms	Illumina GAii, 454 GS FLX, Titanium
MIGS-31.2	Sequencing coverage	340.8 × Illumina; 91.0 × pyrosequence
MIGS-30	Assemblers	Newbler version 2.1-PreRelease-4-28-2009-gcc-3.4.6, phrap, Velvet
MIGS-32	Gene calling method	Prodigal 1.4, GenePRIMP
	INSDC ID	CP002244
	Genbank Date of Release	December 29, 2010
	GOLD ID	Gi03961
	NCBI project ID	38025
	Database: IMG-GEBA	2503538005
MIGS-13	Source material identifier	DSM 12885
	Project relevance	Tree of Life, GEBA

### Growth conditions and DNA isolation

*T. marianensis* 7p75a^T^, DSM 12885, was grown in half strength DSMZ medium 514 (Bacto Marine Broth) [35] at 65°C. DNA was isolated from 0.5-1 g of cell paste using MasterPure Gram-positive DNA purification kit (Epicentre MGP04100) following the standard protocol as recommended by the manufacturer, with modification st/LALM for cell lysis as described in Wu *et al*. [33].

### Genome sequencing and assembly

The genome was sequenced using a combination of Illumina and 454 sequencing platforms. All general aspects of library construction and sequencing can be found at the JGI website [42]. Pyrosequencing reads were assembled using the Newbler assembler version 2.1-PreRelease-4-28-2009-gcc-3.4.6-threads (Roche). The initial Newbler assembly consisting of 30 contigs was converted into a phrap assembly by making fake reads from the consensus, collecting the read pairs in the 454 paired end library. Illumina GAii sequencing data (969.0 Mb) was assembled with Velvet [36] and the consensus sequences were shredded into 1.5 kb overlapped fake reads and assembled together with the 454 data. The 454 draft assembly was based on 216.6 MB 454 draft data and all of the 454 paired end data. Newbler parameters are -consed -a 50 -l 350 -g -m -ml 20. The Phred/Phrap/Consed software package [43] was used for sequence assembly and quality assessment in the following finishing process. After the shotgun stage, reads were assembled with parallel phrap (High Performance Software, LLC). Possible mis-assemblies were corrected with gapResolution [42], Dupfinisher, or sequencing cloned bridging PCR fragments with subcloning or transposon bombing (Epicentre Biotechnologies, Madison, WI) [37]. Gaps between contigs were closed by editing in Consed, by PCR and by Bubble PCR primer walks (J.-F.Chang, unpublished). A total of 132 additional reactions and 4 shatter libraries were necessary to close gaps and to raise the quality of the finished sequence. Illumina reads were also used to correct potential base errors and increase consensus quality using a software Polisher developed at JGI [38]. The error rate of the completed genome sequence is less than 1 in 100,000. Together, the combination of the Illumina and 454 sequencing platforms provided 431.8 × coverage of the genome. Final assembly contained 689,185 pyrosequence and 26,930,845 Illumina reads.

### Genome annotation

Genes were identified using Prodigal [39] as part of the Oak Ridge National Laboratory genome annotation pipeline, followed by a round of manual curation using the JGI GenePRIMP pipeline [40]. The predicted CDSs were translated and used to search the National Center for Biotechnology Information (NCBI) nonredundant database, UniProt, TIGR-Fam, Pfam, PRIAM, KEGG, COG, and InterPro databases. Additional gene prediction analysis and functional annotation was performed within the Integrated Microbial Genomes - Expert Review (IMG-ER) platform [41].

## Genome properties

**Figure 3:**
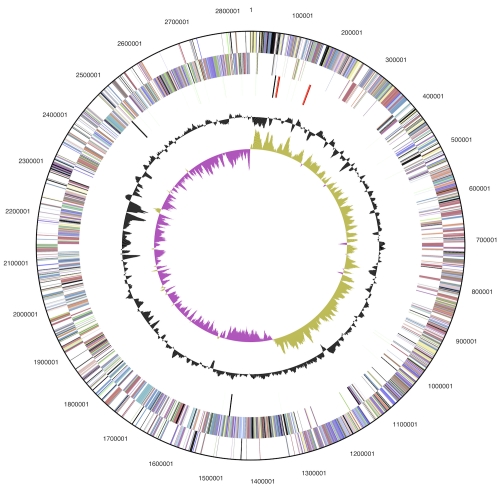
Graphical circular map of the chromosome. From outside to the center: Genes on forward strand (color by COG categories), Genes on reverse strand (color by COG categories), RNA genes (tRNAs green, rRNAs red, other RNAs black), GC content, GC skew.

The genome consists of a 2,844,696 bp long chromosome with a GC content of 72.5% (Table 3 and Figure 3). Of the 2,435 genes predicted, 2,375 were protein-coding genes, and 60 RNAs; 48 pseudogenes were also identified. The majority of the protein-coding genes (74.1%) were assigned with a putative function while the remaining ones were annotated as hypothetical proteins. The distribution of genes into COGs functional categories is presented in Table 4.

**Table 3:** Genome Statistics

**Attribute**	Value	% of Total
Genome size (bp)	2,844,696	100.00%
DNA Coding region (bp)	2,412,792	84.82%
DNA G+C content (bp)	2,061,895	72.48%
Number of replicons	1	
Extrachromosomal elements	0	
Total genes	2,435	100.00%
RNA genes	60	2.46%
rRNA operons	2	
Protein-coding genes	2,375	97.54%
Pseudo genes	48	1.97%
Genes with function prediction	1,804	74.09%
Genes in paralog clusters	288	11.83%
Genes assigned to COGs	1,870	76.80%
Genes assigned Pfam domains	1,996	81.56%
Genes with signal peptides	737	30.27%
Genes with transmembrane helices	647	26.57%
CRISPR repeats	2	

**Table 4:** Number of genes associated with the general COG functional categories

**Code**	**value**	**% age**	**Description**
J	140	6.8	Translation, ribosomal structure and biogenesis
A	0	0.0	RNA processing and modification
K	126	6.1	Transcription
L	99	4.8	Replication, recombination and repair
B	0	0.0	Chromatin structure and dynamics
D	29	1.4	Cell cycle control, cell division, chromosome partitioning
Y	0	0.0	Nuclear structure
V	34	1.7	Defense mechanisms
T	79	3.8	Signal transduction mechanisms
M	101	4.9	Cell wall/membrane/envelope biogenesis
N	49	2.4	Cell motility
Z	0	0.0	Cytoskeleton
W	0	0.0	Extracellular structures
U	49	2.4	Intracellular trafficking, secretion, and vesicular transport
O	73	3.5	Posttranslational modification, protein turnover, chaperones
C	133	6.4	Energy production and conversion
G	108	5.2	Carbohydrate transport and metabolism
E	264	12.8	Amino acid transport and metabolism
F	63	3.1	Nucleotide transport and metabolism
H	96	4.7	Coenzyme transport and metabolism
I	71	3.4	Lipid transport and metabolism
P	108	5.2	Inorganic ion transport and metabolism
Q	44	2.1	Secondary metabolites biosynthesis, transport and catabolism
R	233	11.3	General function prediction only
S	166	8.0	Function unknown
-	565	23.2	Not in COGs
